# Accuracy of Perfusion Index and Perfusion Index Ratio as a Predictor of a Successful Low Interscalene Brachial Plexus Block: A Prospective Observational Study

**DOI:** 10.31486/toj.24.0119

**Published:** 2025

**Authors:** Anuj Jain, Sanjay Srinivas, Zainab Ahmad, Ashutosh Kaushal, Harish Kumar, Vaishali Waindeskar

**Affiliations:** Department of Anesthesiology, All India Institute of Medical Sciences Bhopal, Bhopal, Madhya Pradesh, India

**Keywords:** *Anesthetics–local*, *brachial plexus block*, *perfusion index*, *ROC curve*, *upper extremity*

## Abstract

**Background:**

The perfusion index has previously been used to predict the success of brachial plexus blocks but to our knowledge has not yet been studied for the low interscalene block approach. This study evaluated the accuracy of the perfusion index and perfusion index ratio in predicting the success of a low interscalene brachial plexus block in patients undergoing upper limb surgery.

**Methods:**

The study included 70 patients undergoing upper limb surgeries under ultrasound-guided low interscalene brachial plexus block. After local anesthetic injection, patients’ sensory and motor blocks were assessed every 5 minutes. The perfusion index was recorded at baseline and then at 5-minute intervals until 30 minutes after anesthetic injection in both the blocked and unblocked limbs. The perfusion index ratio (perfusion index at a specific timepoint/baseline perfusion index) was calculated and recorded at each 5-minute time point. Receiver operating characteristic curves were constructed to determine the accurate value of the perfusion index that indicated block success.

**Results:**

The mean perfusion index and perfusion index ratio were higher in the blocked limb vs the unblocked limb at all time points. At the 10-minute time point, the cutoff values for the perfusion index and perfusion index ratio for a successful block were 3.24 and 3.54, respectively. At the 10-minute time point, the sensitivities of the perfusion index and perfusion index ratio in predicting a successful block were 95.2% and 100%, respectively; specificity was 100% for both the perfusion index and perfusion index ratio.

**Conclusion:**

The perfusion index and perfusion index ratio can accurately predict the success of a low interscalene brachial plexus block well before surgical anesthesia is fully established, thereby saving operating room time. A perfusion index ratio of 3.54 or more at 10 minutes is a reliable predictor of block success.

## INTRODUCTION

Brachial plexus block, a conduction block of the sensory, motor, and autonomic nerves of the upper limb, is used to provide surgical anesthesia for upper limb surgeries. The autonomic nerve block precedes the sensory and motor nerve blocks and can result in vasodilation of vessels of the upper limb. This vasodilation manifests as a change in the perfusion index. The perfusion index is the ratio between the pulsatile and the nonpulsatile components of the blood flow in a vessel. As the autonomic nerve block progresses, vessel dilation increases, initially raising the pulsatile fraction of blood and the perfusion index. However, beyond a certain point, blood flow becomes laminar, reducing the difference between pulsatile and nonpulsatile flow, which causes the perfusion index to decrease.^1-3^ The perfusion index has been previously used to predict the success of brachial plexus block but to our knowledge has not been studied for the low interscalene block approach.

Traditionally, brachial plexus block success is assessed by evaluating sensory and motor function in the nerve territory, a subjective and slow process requiring patient cooperation. This method typically takes 20 to 30 minutes, delaying further planning until block success or failure is confirmed.^1-5^ The perfusion index is an objective method of assessment that can help predict block success or failure and save operating room time. However, because the perfusion index after a brachial plexus block depends on the ratio of pulsatile to nonpulsatile blood flow—a value that changes over time—cutoff values that predict block success must be defined at specific time points.

The purpose of this study was to evaluate the accuracy of the perfusion index as an indicator of a successful ultrasound-guided low interscalene brachial plexus block. The primary objective was to determine the perfusion index cutoff value—at 10 minutes after low interscalene brachial plexus block placement—with the best sensitivity and specificity for predicting block results at 30 minutes. The secondary objective was to determine the perfusion index ratio cutoff value—at 10 minutes after low interscalene brachial plexus block placement—with the best sensitivity and specificity for predicting block results at 30 minutes. We hypothesized that the sensitivity and specificity of the perfusion index in predicting a successful block would be >50%.

## METHODS

This prospective observational study was conducted at a tertiary care teaching hospital in central India between September 2021 and February 2023. Approval from the institutional ethical committee, All India Institute of Medical Sciences Bhopal Institutional Human Ethics Committee-Post Graduate Research (IHEC-PGR), was obtained under reference number IHEC-PGR/2021/PG/Jan/01 on August 28, 2021. Written informed consent was obtained from patients for participation in the study and for the use of their data for research and educational purposes. The study was carried out in accordance with the principles of the Declaration of Helsinki (2013), and good clinical practice and ethical principles were followed.

Adult patients who were undergoing unilateral upper limb surgeries under a low interscalene brachial plexus block, who were American Society of Anesthesiologists (ASA) physical status classification 1 or 2, and who had a positive Allen test were included in the study.

Excluded from the study were patients <18 years or >65 years; those unwilling to undergo regional anesthesia; and those having an amputated hand or digit, an arteriovenous fistula or malformation, dysfunctional hemoglobin conditions such as sickle cell anemia, thalassemia, or methemoglobinemia, or long-standing diabetes or hypertension.

Patients were advised to remove nail polish or artificial fingernails (if present) and to avoid applying any local cream. Before the patient was taken to the operating room, both arms were wrapped in cotton sheets of nearly equal thickness to maintain similar temperatures in both limbs and to minimize the effect of ambient cold on peripheral perfusion. Premedication was administered with intravenous midazolam and ondansetron after attaching standard ASA monitors.

Treatment was administered per the usual protocol without any study-specific alterations. In accordance with the technique described by Park et al,^5^ the target area for needle insertion was approximately in the lower third of the interscalene groove. For placement of the brachial plexus block, a Sonosite M-Turbo (FUJIFILM Sonosite, Inc) ultrasonography probe (5-13 MHz) was placed over the supraclavicular region in a transverse oblique orientation, and a supraclavicular view of the subclavian artery and brachial plexus was obtained. The plexus was then traced up the neck until the upper and middle trunks of the plexus were visualized as hypoechoic structures between the anterior and middle scalene muscles in the interscalene groove, recognized as the stoplight sign.

A 22-gauge, 5-cm Stimuplex needle (B. Braun Medical Inc) was advanced using an in-plane approach in a lateral-to-medial direction. The local anesthetic solution was injected below the C6 root of the upper trunk, ensuring uniform drug spread around the visible neural structures. The block was performed by injecting 30 mL of local anesthetic solution (15 mL of 0.5% bupivacaine + 15 mL of 2% lidocaine with adrenaline) under ultrasound guidance.

After needle withdrawal, the surgical limb was evaluated at 5-minute intervals for sensory and motor block for a duration of 30 minutes. The nerve territories of the musculocutaneous, median, radial, and ulnar nerves were assessed for sensory and motor block at each time point. The degree of block at each time point was documented as a composite score.

Sensory blockade of the musculocutaneous nerve (lateral part of the forearm), median nerve (palmar surface of the second finger), radial nerve (dorsal surface of the hand between the thumb and second finger), and ulnar nerve (fifth finger) was graded according to a 3-point scale using a blunt-tip needle pinprick test. Scores were assigned to each area at 5-minute intervals. A score of 0 indicated no sensory block, normal sensation, and pain on pinprick; a score of 1 indicated a partial sensory block with reduced sensation compared to the opposite limb; and a score of 2 indicated a complete sensory block, with complete absence of pain sensation on pinprick.

Motor blockade of the musculocutaneous nerve (elbow flexion), median nerve (arm pronation, wrist flexion, and thumb opposition), radial nerve (elbow extension, wrist extension, and thumb abduction), and ulnar nerve (fourth and fifth finger flexion) was also graded using a 3-point scale. A score of 0 indicated normal movement, a score of 1 indicated paresis (partial block), and a score of 2 indicated complete absence of movement.

Total composite scores were calculated by adding the sensory and motor scores for all areas at specified time intervals. The maximum attainable score was 16. A composite score ≥14 was considered a successful block, while a composite score <14 was considered a block failure.

For the primary outcome measure, a pulse oximeter probe from the BeneView T5 monitor (Shenzhen Mindray Bio-Medical Electronics Co, Ltd) was used to measure perfusion index. Recordings were taken at baseline (before the block was administered) and at 5, 10, 15, 20, 25, and 30 minutes after the local anesthetic injection in both the blocked and unblocked limbs.

For the secondary outcome measure, the perfusion index ratio was calculated as the ratio of the perfusion index at each time point after the injection of local anesthetic to the baseline perfusion index in the same limb.

Based on the study by Abdelnasser et al,^1^ the sample size was calculated to detect an area under the receiver operating characteristic (AUROC) curve of 0.8, with a null hypothesis AUROC curve of 0.5. Considering a block failure rate of 10%, a sample size of 70 patients was required to achieve a study power of 80% and an α error of 0.05.

Data were analyzed using SPSS Statistics version 16.0 (IBM Corporation). Data were assessed for normality using the Kolmogorov-Smirnov test. Parametric tests were used for normally distributed data, and nonparametric tests were used for nonnormally distributed data. Perfusion index and perfusion index ratio, measured at various time points and normally distributed, were analyzed using parametric tests. Means and standard deviations were calculated, along with 95% confidence intervals. The significance of perfusion index and perfusion index ratio data at various time points was analyzed using the independent sample *t* test and paired sample *t* test. The receiver operating characteristic curve was plotted for the perfusion index and perfusion index ratio at each time point against the binary outcome, the total composite score of the block at the 30-minute time point, to determine the values of the perfusion index and perfusion index ratio with the best sensitivity and specificity at each time point. The area under the curve (AUC) and cutoff values for the perfusion index and perfusion index ratio were analyzed accordingly.

A 2-way repeated measures analysis of variance (ANOVA) was used to assess the effects of time and sex on the measured perfusion index and perfusion index ratio values.

## RESULTS

A total of 70 patients underwent upper limb surgery under the effect of a low interscalene brachial plexus block. Demographic and clinical characteristics of the study population are presented in Table 1. The majority of patients were male (72.9%); most patients were aged 18 to 45 years (75.7%). Most surgeries were in the elbow, below elbow, and wrist and hand areas (88.6%), with the majority of patients (81.4%) undergoing open reduction and internal fixation, and only 18.6% undergoing superficial surgeries such as skin debridement.

**Table 1.** Demographic and Clinical Characteristics of the Study Population

Variable	All Patients, n=70
Age, years	
18 to 25	19 (27.1)
26 to 35	16 (22.9)
36 to 45	18 (25.7)
46 to 55	10 (14.3)
>55	7 (10.0)
Sex	
Male	51 (72.9)
Female	19 (27.1)
Location of surgery	
Shoulder	2 (2.9)
Above elbow	6 (8.6)
Elbow and below elbow	42 (60.0)
Wrist and hand	20 (28.6)
Type of procedure	
Skin	13 (18.6)
Skin and bone	57 (81.4)
Nature of surgery	
Elective	46 (65.7)
Emergency	24 (34.3)
Side of surgery	
Right	40 (57.1)
Left	30 (42.9)

Note: Data are presented as n (%).

Because the baseline perfusion index was close to unity, the perfusion index and perfusion index ratio values were very similar. In the blocked limbs, the perfusion index and perfusion index ratio showed a consistent increase until the 15-minute time point, after which they began to decline (Table 2). At all time points, the perfusion index and perfusion index ratio were significantly higher in the blocked limbs compared to the unblocked limbs.

**Table 2.** Mean Perfusion Index and Perfusion Index Ratio Values at All Time Points in the Blocked vs Unblocked Limbs, n=70

Time Point, minutes	Variable	Blocked Limb	Unblocked Limb	*P* Value
Baseline	Perfusion index	1.03 ± 0.41 (0.934, 1.126)	1.04 ± 0.40 (0.946, 1.134)	0.9
5	Perfusion index	3.91 ± 1.91 (3.463, 4.357)	1.08 ± 0.42 (0.982, 1.178)	**<0.001**
	Perfusion index ratio	3.89 ± 1.44 (3.553, 4.227)	1.03 ± 0.07 (1.014, 1.046)	**<0.001**
10	Perfusion index	6.80 ± 3.02 (6.093, 7.507)	1.06 ± 0.40 (0.966, 1.154)	**<0.001**
	Perfusion index ratio	6.83 ± 2.40 (6.268, 7.392)	1.02 ± 0.10 (0.997, 1.043)	**<0.001**
15	Perfusion index	7.18 ± 3.32 (6.402, 7.958)	1.07 ± 0.42 (0.972, 1.168)	**<0.001**
	Perfusion index ratio	7.19 ± 2.54 (6.595, 7.785)	1.03 ± 0.09 (1.009, 1.051)	**<0.001**
20	Perfusion index	5.10 ± 2.42 (4.533, 5.667)	1.08 ± 0.41 (0.984, 1.176)	**<0.001**
	Perfusion index ratio	5.09 ± 1.86 (4.654, 5.526)	1.03 ± 0.08 (1.011, 1.049)	**<0.001**
25	Perfusion index	4.70 ± 1.98 (4.236, 5.164)	1.08 ± 0.41 (0.984, 1.176)	**<0.001**
	Perfusion index ratio	4.65 ± 1.96 (4.191, 5.109)	1.03 ± 0.08 (1.011, 1.049)	**<0.001**
30	Perfusion index	3.55 ± 1.98 (3.086, 4.014)	1.07 ± 0.42 (0.972, 1.168)	**0.004**
	Perfusion index ratio	3.50 ± 1.43 (3.165, 3.835)	1.03 ± 0.09 (1.009, 1.051)	**<0.001**

Notes: Data are presented as mean ± SD (95% CI). Significant *P* values are in bold.

**Figure 1.**
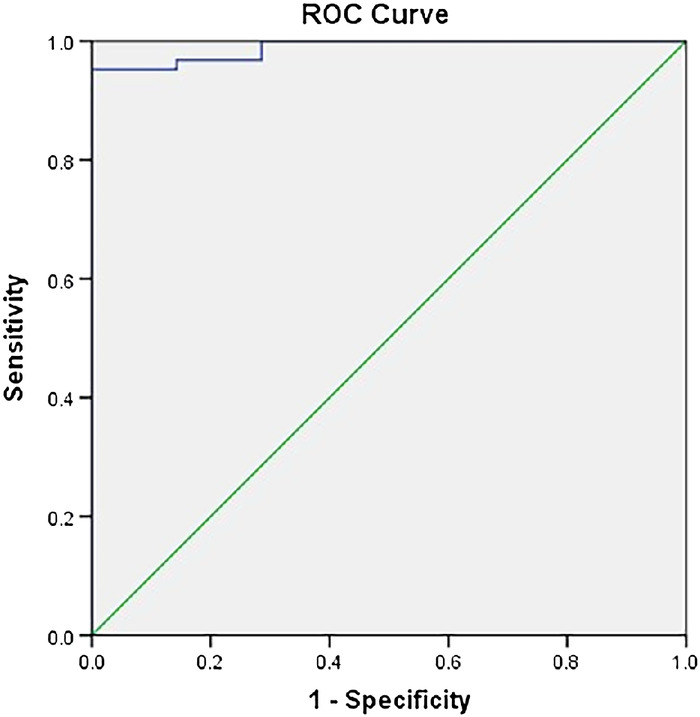
Receiver operating characteristic (ROC) curve plotted between the perfusion index and the total composite score at the 10-minute time point. The area under the curve at the 10-minute time point is 0.989. The cutoff value of the perfusion index is 3.24, with a best sensitivity of 95.2% and a specificity of 100%.

**Figure 2.**
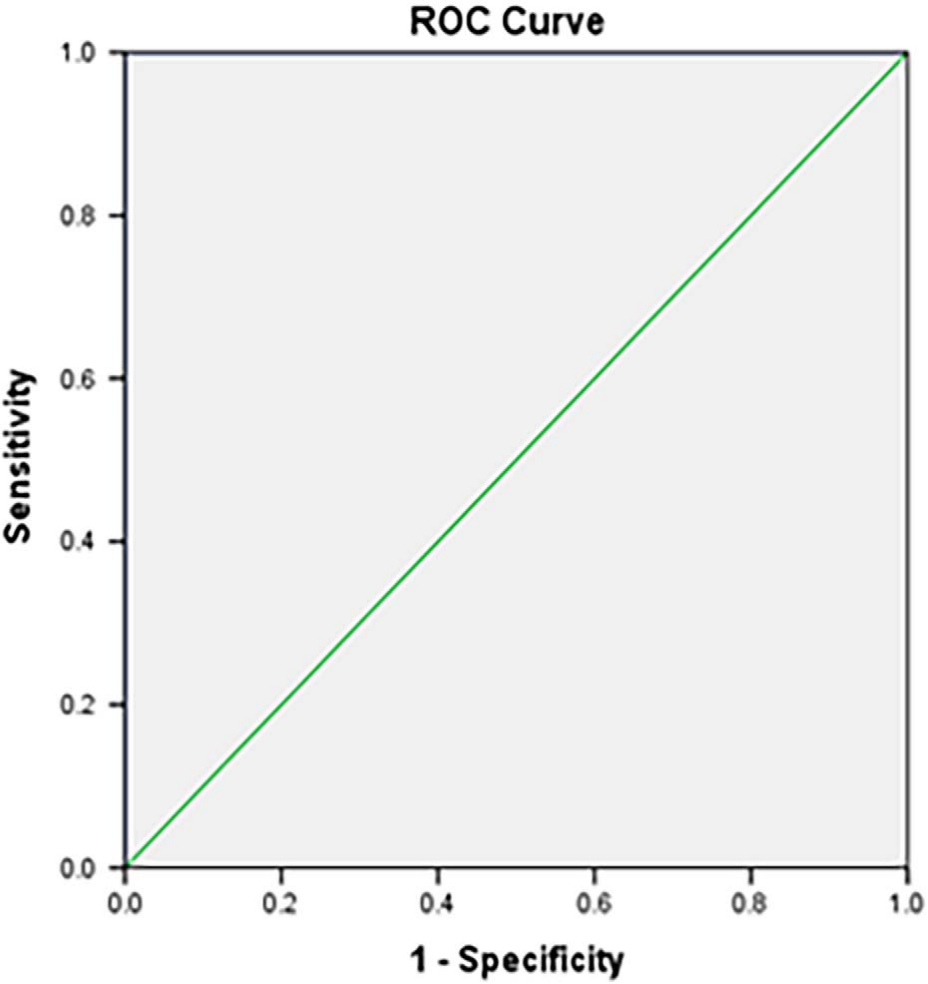
Receiver operating characteristic (ROC) curve plotted between the perfusion index ratio and the total composite score at the 10-minute time point. The area under the curve at the 10-minute time point is 1.000. The cutoff value of the perfusion index ratio is 3.54, with a best sensitivity of 100% and a specificity of 100%.

At the 30-minute time point, 63 of 70 patients had a composite score ≥14. For the perfusion index, the AUC at 10 minutes was 0.989. The cutoff value of the perfusion index was 3.24, with a sensitivity of 95.2% and a specificity of 100% (Figure 1). For the perfusion index ratio, the AUC was 1.000. The cutoff value of the perfusion index ratio was 3.54, with a sensitivity of 100% and a specificity of 100% (Figure 2).

At the 10-minute time point, the incidence of incomplete sensory block ranged from 25.7% to 40%, while incomplete motor block was observed in 70% to 84% of patients. At the 30-minute time point, almost 90% of patients had developed a successful block (Table 3).

**Table 3.** Incidence of Patients With an Incomplete Block at the 10-Minute Time Point and a Complete Block at the 30-Minute Time Point, n=70

	10-Minute Time Point		30-Minute Time Point	
Nerve Territory	Incomplete Sensory Block	Incomplete Motor Block	Complete Sensory Block	Complete Motor Block
Musculocutaneous	18 (25.7)	49 (70.0)	63 (90.0)	64 (91.4)
Median	21 (30.0)	56 (80.0)	63 (90.0)	64 (91.4)
Radial	23 (32.9)	57 (81.4)	63 (90.0)	64 (91.4)
Ulnar	28 (40.0)	59 (84.3)	63 (90.0)	61 (87.1)

Notes: Data are presented as n (%).

The perfusion index and perfusion index ratio were significantly higher in patients with a successful block vs patients with an unsuccessful block (Table 4).

**Table 4.** Mean Perfusion Index and Perfusion Index Ratio Values at All Time Points for Patients With Successful vs Unsuccessful Blocks, n=70

Time Point, minutes	Variable	Successful Block, n=63	Unsuccessful Block, n=7	*P* Value
Baseline	Perfusion index	1.03 ± 0.41 (0.929, 1.131)	1.05 ± 0.41 (0.746, 1.354)	0.9
5	Perfusion index	4.14 ± 1.86 (3.681, 4.599)	1.76 ± 0.73 (1.219, 2.301)	**<0.001**
	Perfusion index ratio	4.14 ± 1.29 (3.821, 4.459)	1.69 ± 0.41 (1.386, 1.994)	**<0.001**
10	Perfusion index	7.34 ± 2.67 (6.681, 7.999)	1.95 ± 0.92 (1.268, 2.632)	**<0.001**
	Perfusion index ratio	7.39 ± 1.80 (6.946, 7.834)	1.84 ± 0.55 (1.433, 2.247)	**<0.001**
15	Perfusion index	7.76 ± 2.96 (7.029, 8.491)	1.96 ± 0.74 (1.412, 2.508)	**<0.001**
	Perfusion index ratio	7.78 ± 1.93 (7.303, 8.257)	1.96 ± 0.62 (1.501, 2.419)	**<0.001**
20	Perfusion index	5.47 ± 2.25 (4.914, 6.026)	1.76 ± 0.72 (1.227, 2.293)	**<0.001**
	Perfusion index ratio	5.47 ± 1.56 (5.085, 5.855)	1.76 ± 0.66 (1.271, 2.249)	**<0.001**
25	Perfusion index	4.70 ± 1.96 (4.216, 5.184)	1.67 ± 0.81 (1.070, 2.270)	**<0.001**
	Perfusion index ratio	4.56 ± 1.90 (4.091, 5.029)	1.59 ± 0.77 (1.020, 2.160)	**<0.001**
30	Perfusion index	3.77 ± 1.96 (3.286, 4.254)	1.54 ± 0.54 (1.140,1.940)	**0.004**
	Perfusion index ratio	3.72 ± 1.34 (3.389, 4.051)	1.58 ± 0.64 (1.106, 2.054)	**<0.001**

Note: Data are presented as mean ± SD (95% CI). Significant *P* values are in bold.

The perfusion index and perfusion index ratio in the blocked limb were comparable between male and female subsets of the study population at all time points (Table 5).

**Table 5.** Mean Perfusion Index and Perfusion Index Ratio Values at All Time Points in Males vs Females, n=70

Time Point, minutes	Variable	Males, n=51	Females, n=19	*P* Value
Baseline	Perfusion index	1.09 ± 0.43 (0.972, 1.208)	0.89 ± 0.31 (0.751, 1.029)	0.07
5	Perfusion index	4.15 ± 1.92 (3.623, 4.677)	3.26 ± 1.80 (2.451, 4.069)	0.08
	Perfusion index ratio	3.95 ± 1.33 (3.585, 4.315)	3.74 ± 1.73 (2.962, 4.518)	0.5
10	Perfusion index	7.21 ± 3.06 (6.370, 8.050)	5.70 ± 2.69 (4.490, 6.910)	0.06
	Perfusion index ratio	6.88 ± 2.05 (6.317, 7.443)	6.70 ± 3.21 (5.257, 8.143)	0.7
15	Perfusion index	7.66 ± 3.43 (6.719, 8.601)	5.90 ± 2.65 (4.708, 7.092)	0.04
	Perfusion index ratio	7.28 ± 2.21 (6.673, 7.887)	6.95 ± 3.34 (5.448, 8.452)	0.6
20	Perfusion index	5.43 ± 2.54 (4.733, 6.127)	4.21 ± 1.85 (3.378, 5.042)	0.06
	Perfusion index ratio	5.16 ± 1.68 (4.699, 5.621)	4.93 ± 2.32 (3.887, 5.973)	0.6
25	Perfusion index	4.76 ± 2.12 (4.178, 5.342)	3.87 ± 1.93 (3.002, 4.738)	0.1
	Perfusion index ratio	4.36 ± 3.90 (3.290, 5.430)	4.60 ± 2.12 (3.647, 5.553)	0.6
30	Perfusion index	3.83 ± 2.14 (3.243, 4.417)	2.79 ± 1.25 (2.228, 3.352)	0.05
	Perfusion index ratio	3.60 ± 1.48 (3.194, 4.006)	3.24 ± 1.29 (2.660, 3.820)	0.3

Note: Data are presented as mean ± SD (95% CI).

For perfusion index values, the 2-way repeated measures ANOVA showed a significant main effect of time (F(6, 476)=62.40, *P* <0.001), indicating a significant change over repeated time points. A significant main effect of sex (F(1, 476)=23.74, *P* <0.001) suggested a statistically significant difference between sexes. Additionally, a significant interaction effect between time and sex (F(6, 476)=2.18, *P*=0.044) indicated that the pattern of change over time differed between the sexes.

For perfusion index ratio values, while a significant main effect of time was observed (F(5, 408)=22.21, *P* <0.001), the main effect of sex was not significant (F(1, 408)=1.10, *P*=0.295), suggesting no significant difference in overall means between the sexes. The interaction effect between time and sex was also not significant (F(5, 408)=0.82, *P*=0.539), indicating a similar pattern of change over time in both sexes. The ANOVA findings suggest that while sex differences significantly influenced perfusion index values over time, they did not have a significant impact on perfusion index ratio values or their temporal pattern.

## DISCUSSION

In this study, the cutoff values at the 10-minute time point for the perfusion index and perfusion index ratio for a successful block were 3.24 and 3.54, respectively.

The perfusion index is a simple, noninvasive method for assessing the pulsatility of blood flow. The pulsatile component is influenced by factors such as vasoconstriction and vasodilation. Various factors affect the pulsatile fraction of blood flow, including the extent of sympathetic blockade, vascularity of the limb, intravascular volume status, and temperature.

Lal et al studied the perfusion index in the upper limb after supraclavicular brachial plexus block.^6^ Because different approaches to brachial plexus block vary in the extent of the block (ie, the nerve territories affected), perfusion index values are likely to differ for each approach. To our knowledge, perfusion index values and trends have not been defined for brachial plexus block using the low interscalene block approach. Because the perfusion index is influenced by sympathetic blockade, the extent of sympathetic involvement might differ based on whether the block is higher (interscalene) or lower (supraclavicular). A low interscalene block combines the advantages of the classic interscalene block and the supraclavicular block,^4,5^ reduces the associated complications,^7^ and consequently may become the most popular approach for upper limb surgical anesthesia. With respect to the nerve territories blocked by a low interscalene block, the findings of our study are consistent with those of other studies.^4,5,7^

In this study, we carefully defined our cohort, only including patients without any issues related to vascularity or intravascular volume status. Another methodological improvement we implemented, compared to the study by Lal et al,^6^ was to wrap the patients’ arms in cotton sheets to mitigate the impact of the cold ambient temperature in the operating room. The perfusion index is influenced by peripheral vasodilation, which is affected not only by sympathetic blockade but also by external temperature changes. By wrapping patients’ arms in cotton and ensuring equal insulation, our study eliminated a confounding factor and helped ensure that the perfusion index changes were attributable to the block's effect on vascular tone. This study modification enhanced the accuracy and reliability of the perfusion index measurements.

Another of our methodological improvements was to use the unoperated upper limb as a control. Lal et al measured the perfusion index only in the blocked limb, and any observed changes in perfusion index were assumed to be solely attributable to the block.^6^ However, the perfusion index is not an absolute value; it can fluctuate due to systemic hemodynamic changes (eg, anxiety, sedation, general vasodilation from midazolam, or changes in blood pressure). By including a contralateral control limb, our study accounted for these systemic variations. Consequently, a perfusion index increase in the blocked limb with an unchanged perfusion index in the unblocked limb supports the finding that the perfusion index increase is specific to the block. Our data demonstrated a statistically significant difference in the perfusion index between the blocked and unblocked limbs at all time points after baseline.

In the 2-way repeated measures ANOVA, the perfusion index ratio showed no significant difference between sexes. The significant interaction and between-subject effects observed for the perfusion index suggest inherent differences between males and females in baseline perfusion and its temporal trajectory. However, when the data were normalized to baseline (the perfusion index ratio), these differences were no longer evident, indicating that while the perfusion index varies with sex, the relative physiologic response pattern remains consistent in males and females.

In our study, the perfusion indexes in the blocked and unblocked limbs were close to unity at baseline, a finding that differs from findings in other similar studies.^1,3,8-12^ This aspect makes our study unique and enhances the validity of the observed changes in the perfusion index in the blocked limb.

In our study, the rate of change in the mean perfusion index was greatest up to the 10-minute time point; the highest mean perfusion index value was measured at the 15-minute time point, and the values then decreased at the remaining time points. This finding supports the utility of tracking the perfusion index up to 10 minutes after local anesthetic injection to predict the outcome at 30 minutes.

A higher proportion of patients with incomplete sensory or motor block at the 10-minute time point had developed a complete block at the 30-minute time point. This observation underscores the practical applicability of the perfusion index in minimizing time wastage in the operating room.

We hypothesized that the sensitivity and specificity of the perfusion index in predicting a successful block would be >50%. The AUROC was high, thereby validating the results. Although the specificity at the cutoff value was 100% for both the perfusion index and perfusion index ratio, the sensitivity was higher for the perfusion index ratio (100% vs 95.2%).

Our findings are consistent with a 2017 study by Abdelnasser et al.^1^ In their study of a patient cohort undergoing upper limb surgery under ultrasound-guided supraclavicular nerve block, the baseline mean perfusion index in the study limb was 2.8 ± 0.8. The cutoff values for the perfusion index and perfusion index ratio at the 10-minute time point were quite different from each other at 3.3 and 1.4, respectively, but the sensitivity and specificity were similar to the sensitivity and specificity in our study. In the Abdelnasser et al study, the AUROC was 100%, and the sensitivity and specificity of the perfusion index and perfusion index ratio at the 10-minute time point were 100%.^1^

A perfusion index ratio value is preferable to a perfusion index value as it normalizes the perfusion index value for any differences that may have existed at baseline. Furthermore, using the perfusion index ratio also accounts for variations in results that may arise because of differences in the type of pulse oximeter probe used.

Our study has certain important limitations. First, we included only patients with ASA physical status classification 1 or 2 who did not have any previous vascular or nerve pathology, so the results cannot be generalized. Second, our study had a small sample size.

## CONCLUSION

The perfusion index and perfusion index ratio are reliable predictors of block results after administering a low interscalene brachial plexus block and may help save operating room time. Both the perfusion index and perfusion index ratio tend to peak approximately 15 minutes after local anesthesia injection. The perfusion index ratio seems to be the better indicator of success between the two because it normalizes the value for the baseline value of the perfusion index.

## Notes

*This article meets the Accreditation Council for Graduate Medical Education and the American Board of Medical Specialties Maintenance of Certification competencies for Patient Care, Medical Knowledge, and Practice-Based Learning and Improvement.*
